# Distribution of *Mycoplasma haemofelis* in blood and tissues following experimental infection

**DOI:** 10.1016/j.micpath.2009.09.009

**Published:** 2009-12

**Authors:** Séverine Tasker, Iain R. Peters, Michael J. Day, Barbara Willi, Regina Hofmann-Lehmann, Timothy J. Gruffydd-Jones, Chris R. Helps

**Affiliations:** a School of Clinical Veterinary Science, University of Bristol, Langford, Bristol BS40 5DU, United Kingdom; b Clinical Laboratory, Vetsuisse Faculty, University of Zurich, Zurich, Switzerland

**Keywords:** Haemoplasma, Quantitative real-time PCR, Sequestration, *M. haemofelis*

## Abstract

The aim of the study was to describe blood and tissue copy number distribution during *Mycoplasma haemofelis* infection and determine if sequestration of organisms in body tissues could explain blood copy number cycling in infected cats. Thirteen domestic–shorthaired cats were used. Blood samples were regularly collected, and at a differing time point post-infection for each cat, tissue samples also collected, for quantitative PCR (qPCR). Absolute haemoplasma copy numbers were calculated for all blood and tissue samples, as well as an estimation of the ratio of tissue haemoplasma copy number to that expected in the tissue if a positive qPCR result arose due to tissue blood supply alone. Cats with high or moderate *M. haemofelis* blood copy numbers at the time of tissue collection had fewer *M. haemofelis* copies in most tissues than expected due to the tissue blood supply alone; only splenic and lung tissues consistently contained more *M. haemofelis*. However tissues collected from cats at a time of very low *M. haemofelis* blood copy numbers, when putative copy number cycling nadirs were occurring, were usually qPCR negative. Hence no evidence of significant tissue *M. haemofelis* sequestration was found in this study to explain the copy number cycling reported with this feline haemoplasma species.

## 1 Introduction

The feline haemoplasma species *M. haemofelis* can cause severe haemolytic anaemia [1–3]. The development of quantitative real-time polymerase chain reaction (qPCR) assays for detection of feline haemoplasma DNA [4–9] has allowed successful monitoring of the kinetics of blood haemoplasma copy number in experimentally infected cats. Studies have found that the maximum copy number following infection with *M. haemofelis* occurs around 14–15 days post-infection (DPI) (10^8.6^–10^9.6^ copies/ml of blood) but with marked variation in *M. haemofelis* copy number over time [4,7,9,10]. These fluctuations in *M. haemofelis* copy number can be as large and as rapid as a 4 log difference over 2 or 3 days, or a 7 log difference over 12 days. Further investigation is required to elucidate the mechanisms behind the copy number variation seen in *M. haemofelis-*infected cats. Possibilities include sequestration and subsequent release of organisms from organs such as the spleen, liver or lungs [11], rapid removal and subsequent rapid multiplication of any remaining organisms in the blood, or the effect of cyclical antigenic variation used by some haemotropic and mycoplasmal species to evade the host immune response [12,13].

The aim of the current study was to describe the blood and tissue *M. haemofelis* copy number distribution during experimental infection and determine if sequestration of organisms in body tissues could explain blood copy number cycling in infected cats. Monitoring was performed by measuring haemoplasma copy numbers within the circulation and target tissues.

## 2 Materials and methods

### 2.1 Study design and protocol

Thirteen barrier-maintained specific pathogen free derived domestic–shorthaired cats were used. Table 1 shows a summary of the cats used and inoculations given. The discontinuous numbering of the cats was due to the inclusion of additional cats in a parallel study documenting haematological, Coombs' test and blood glucose concentrations in haemoplasma-infected cats [10]. All procedures and experiments described were undertaken under a project license approved under the UK Animals (Scientific Procedures) Act 1986.

Experimental infection of the cats with *M. haemofelis* was carried out by obtaining heparinised blood from a barrier-maintained donor cat chronically infected with *M. haemofelis*. The blood type of the donor cat and all recipients was predetermined to be Type A (RapidVet-H blood typing cards, DMS Laboratories Inc, New Jersey, US). Following collection of fresh blood from the donors, the heparinised blood was placed on wet ice and 2 ml of heparinised donor blood was given intravenously to each cat via pre-placed cephalic intravenous catheters, within 5 min of collection (Table 1). All cats were inoculated with the same isolate of *M. haemofelis*.

Blood samples were collected from all cats three times weekly for packed cell volume (PCV) determination and haemoplasma copy number qPCR. Cats were monitored daily for signs of ill health and were given subcutaneous fluid therapy (lactated Ringer's solution, up to 100 ml/kg/day) if they appeared pale, dehydrated or were anorexic. If the PCV fell to ≤ 10%, doxycycline at 10 mg/kg/day PO (followed by 5 ml water by syringe PO) was given until it rose above 10%.

Samples of tissue were collected from the cats immediately following euthanasia with an overdose of pentobarbiturate anaesthesia, and immediately after blood sample collection to enable comparison of blood and tissue *M. haemofelis* copy numbers contemporaneously. The tissue samples were collected at various DPI in different cats to enable comparison of blood and tissue *M. haemofelis* copy numbers at different stages of infection. Blood copy numbers were defined as being high if > 10^9^ copies/ml blood, moderate if between 10^7^ and 10^9^ copies/ml blood inclusive, and low if < 10^7^ copies/ml blood. Tissues obtained from cats at times of low blood copy numbers were either collected as soon as, or very soon after, a single negative blood *M. haemofelis* qPCR result was obtained (Cats HF2, HF4, HF8 and HF12), or during the nadir of a putative copy number cycle in the blood (Cat HF6) to look for evidence of *M. haemofelis* tissue sequestration. Since the negative qPCR results obtained in Cats HF2, HF8 and HF12 followed variable degrees of blood haemoplasma copy number cycling, tissues collected in these cats also allowed investigation of possible tissue sequestration of *M. haemofelis*.

Tissues collected were tonsil, submandibular lymph node, bone marrow, lung, liver, spleen, kidney cortex, kidney medulla, jejunum, colon, mesenteric lymph node and colonic lymph node. Each tissue sample was collected with new disposable scalpels and forceps to avoid cross contamination between tissues. Samples were divided and one half was placed in 10% neutral–buffered formalin and processed routinely for histopathological examination of haematoxylin and eosin stained sections. The second half was placed immediately into at least 10 volumes of RNAlater RNA stabilization reagent (Qiagen, Crawley, UK), and stored at −4 °C for 24 h. After 24 h the RNAlater reagent was decanted and the tissue samples then stored at −80 °C.

### 2.2 DNA extraction

For blood samples, DNA was extracted from 100 μl of EDTA-anticoagulated blood using the Macherey-Nagel Nucleospin Blood kit (ABgene, Epsom, UK), eluting into 100 μl of Buffer BE. DNA from blood samples from known *M. haemofelis* non-infected and infected cats were concurrently extracted with each batch of 24 samples as negative and positive controls respectively. For tissue samples, 10 mg of each tissue was used for DNA extraction. Each tissue was subjected to overnight lysis by incubation with 180 μl tissue lysis buffer (Buffer ATL, Qiagen, Crawley, UK) and 20 μl proteinase K on a shaker (1000 rpm) at 56 °C. Following this, 200 μl of lysis buffer (Buffer BQ1, Macherey-Nagel Nucleospin Blood kit, ABgene, Epsom, UK) was added to each sample which was then incubated on a shaker (1000 rpm) at 70 °C for 10 min. Thereafter, the Nucleospin Blood kit (ABgene, Epsom, UK) protocol was followed (from the ethanol addition step) for DNA extraction from the tissues, again eluting into 100 μl of Buffer BE.

### 2.3 PCR assays

*M. haemofelis* qPCR assay (incorporating a duplex feline 28S rDNA assay as an internal control) was carried out as previously described [8]. This qPCR incorporates *M. haemofelis* specific primers (forward 5′-GTGCTACAATGGCGAACACA-3′ and reverse 5′- TCCTATCCGAACTGAGACGAA-3′) and *Taq*man probe (FAM- TGTGTTGCAAACCAGCGATGGT- Black hole quencher [BHQ]1), and feline 28S rDNA specific primers (forward 5′-AGCAGGAGGTGTTGGAAGAG 3′ and reverse 5′-AGGGAGAGCCTAAATCAAAGG-3′) and *Taq*man probe (Texas Red-TGGCTTGTGGCAGCCAAGTGT-BHQ2). QPCRs were performed using Qiagen HotStarTaq Master Mix (Crawley, UK) with 200 nM of each primer, 100 nM of each probe, 4.5 mM MgCl_2_ and 5 μl of DNA template in a total volume of 25 μl. QPCRs were performed in an iCycler IQ (Bio-Rad Laboratories Ltd, Hemel Hempstead, UK) with an initial incubation of 95 °C for 15 min and 45 cycles of 95 °C for 10 s and 60 °C for 30 s during which the fluorescence data were collected.

Blood DNA extraction and qPCR analyses were performed on the days of blood collection whilst tissue samples were analysed up to 6 months following collection and storage. DNA samples from known *M. haemofelis*-infected cats and water were subjected to qPCR as positive and negative controls respectively. Quantification of *M. haemofelis* copy number in each qPCR was derived by comparison to a standard curve generated by amplification of plasmids containing cloned 16S rDNA *M. haemofelis* PCR products, as described previously [4,5]. The 28S rDNA qPCR results were used as internal controls to assure quality control but were not used to standardize haemoplasma copy numbers between cats or tissues.

### 2.4 Analysis of results

Tissue *M. haemofelis* copy numbers were compared to blood haemoplasma copy numbers in each cat on the same DPI. To allow meaningful comparisons allowances were made since the starting material used for DNA extraction differed for blood (100 μl) and tissue samples (10 mg). It was assumed that the qPCR copy numbers obtained for the blood samples were the equivalent of those present in 5 μl of blood (since 5 μl of 100 μl DNA prepared from 100 μl of blood was used in the qPCR) whilst those obtained for the tissue samples were equivalent to those present in 0.5 mg of tissue (since 5 μl of 100 μl DNA prepared from 10 mg of tissue was used in the qPCR). The qPCR results were expressed as copy numbers per ml of blood by multiplying the results by 200. Additionally, an allowance had to be made for the presence of blood within the tissue samples. This was approximated using the estimated blood volume (66 ml/kg) of a normal cat [14] to derive blood volume present at the time of tissue collection as well as taking into account the volume of blood samples collected immediately before tissue collection. It was necessary to assume that blood volume was equally distributed amongst all tissues since published tissue blood volume values for individual tissues were not available to allow refinement of this calculation. These calculations, together with the *M. haemofelis* copy number found in the blood of the cat at the time of tissue collection, were used to determine how many *M. haemofelis* copies would be expected to be found in a tissue sample, if the tissues were PCR positive due to the presence of blood in the tissue alone. These allowances allowed the identification of tissue types that broadly contained more, less or equivalent *M. haemofelis* copies than would be accounted for by the blood supply to those tissues alone.

## 3 Results

All cats were *M. haemofelis* qPCR negative on Day 0 before inoculation. Positive qPCR results were first obtained at 2 DPI for most cats except for HF13 (positive at 1 DPI). All blood samples gave acceptable 28S rDNA qPCR results (threshold cycle [C_T_] values of 26.9–29.1) indicating the presence of amplifiable DNA in all samples and an absence of significant PCR inhibitors. All but one tissue sample gave acceptable 28S rDNA qPCR results (C_T_s of 25.1–30.9). One of the tissue samples (HF3 bone marrow) initially was negative on 28S rDNA qPCR but repeat analysis with another aliquot of the same extracted DNA preparation gave a positive result (C_T_ 27.2) allowing inclusion of the corresponding haemoplasma qPCR result.

**Fig. 1:**
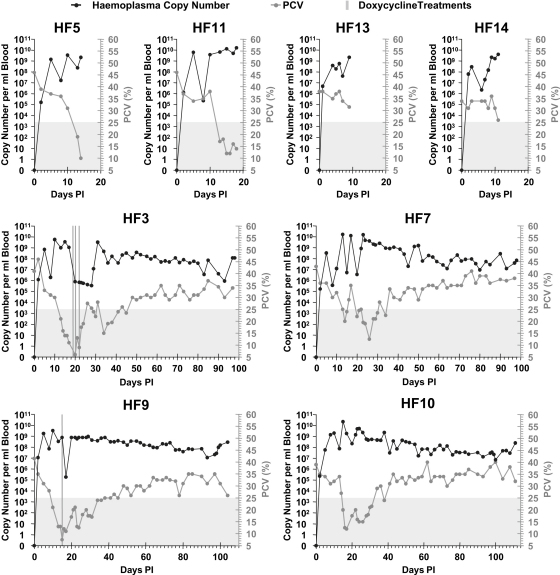
*M. haemofelis* blood copy number against days post-infection (PI) for cats which had high or moderate blood copy numbers at the time of tissue collection. PCV values are also plotted against time, with the grey shaded area representing anaemia. Days on which oral doxycycline treatment (10 mg/kg/day) was given are indicated by vertical lines.

**Fig. 2:**
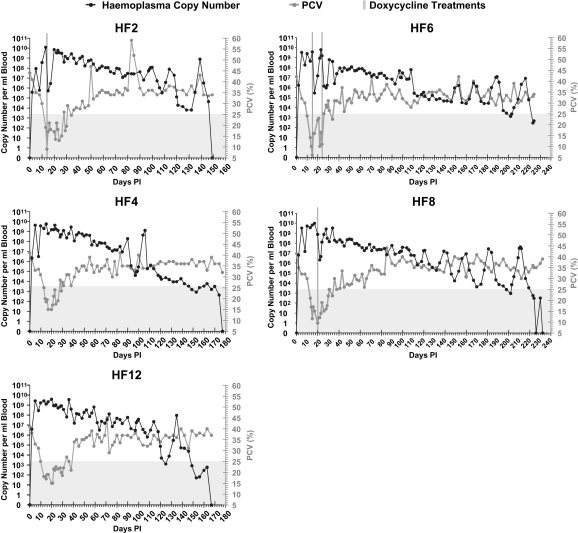
*M. haemofelis* blood copy number against days PI for cats which had low blood copy numbers at the time of tissue collection. PCV values are also plotted against time, with the grey shaded area representing anaemia. Days on which oral doxycycline treatment (10 mg/kg/day) was given are indicated by vertical lines.

Figs. 1 and 2 show *M. haemofelis* blood copy number and PCVs against time. All cats, other than HF13 and HF14 which were only monitored for a short period, developed anaemia. Generally, the lowest PCV recorded in those cats that were monitored throughout the period of anaemia coincided with, or just followed, peak *M. haemofelis* copy numbers.

Although some marked copy number variation was seen early in the course of *M. haemofelis* infection, particularly in some cats (e.g. HF6 and HF7), longer term copy number cycling was not frequently seen despite the long follow up period in many cats. Cats HF8 and HF6 showed some evidence of long term repeated cycling after 114 and 146 DPI respectively. These cycles involved up to a 6 log change in *M. haemofelis* copy number and lasted between 14 and 32 days, the cycles tending to increase in duration with subsequent cycles. Cat HF12 also showed some evidence of marked variation in *M. haemofelis* copy number towards the end of the monitoring period, but since only one, or possibly two cycles, were recorded, it is hard to be sure that these variations constituted true copy number cycling.

Table 2 shows that in cats with high or moderate numbers of *M. haemofelis* in their blood at the time of tissue collection, most tissues actually contained far fewer *M. haemofelis* copies (i.e. ratio < 1) than would be expected based on the estimate of copies present due to blood within the tissues. Those tissues that tended to contain many more *M. haemofelis* copies compared to that estimated due to blood supply alone (ratios ≥ 5) were the spleen (6/8 cats) and lung (5/8 cats).

For the four cats which had tissues collected at times when the blood *M. haemofelis* qPCR was negative (HF2, HF4, HF8 and HF12), repeat qPCR analysis was performed eight times using replicate aliquots of the same extracted DNA preparations to assess repeatability of the negative blood qPCR result and obtain an average *M. haemofelis* blood copy number for each of these cats at the time of tissue collection, as shown in Table 2. These cats, along with HF6 (who had samples collected at a putative copy number cycle nadir) showed very few tissue positive qPCR results for *M. haemofelis*, despite the presence of adequate amounts of 28S rDNA. However, although the positive results that were obtained for the tissues showed the presence of only low copy numbers of *M. haemofelis*, the numbers present were greater than expected due to the tissue blood supply alone, with ratios of 27 to 6180.

Microscopic examination of the tissues collected from the cats showed no significant pathology other than changes consistent with severe anaemia including centrilobular hepatocyte anoxic changes and bone marrow erythroid hyperplasia (data not shown).

## 4 Discussion

The present study is the first to document haemoplasma copy numbers in tissues collected from haemoplasma-infected cats and to attempt to determine whether the infectious load in some tissues may proportionally differ from that in contemporaneously collected blood. By this process we aimed to identify which body tissues might act as sites of sequestration of *M. haemofelis* during the nadir of cycles of blood copy number.

A fully standardized approach to inoculum dose and recipient cat signalment would have been preferable to allow more direct comparisons between cats. However, this was not possible due to practical issues regarding cat availability and inoculum preservation. Moreover tissues had to be collected at different time points making monitoring periods differ greatly between cats. The lack of an *in vitro* culture method for *M. haemofelis* makes it very difficult to obtained standardized inocula to use in experimental studies unless several cats are infected simultaneously from the same carrier cat, as was performed for 11 of the cats in the current study. Studies have not been performed investigating the effect of inoculum dose, age or sex on the outcome of haemoplasma infection although observational studies carried out in a parallel study [10] suggested that younger cats may be more likely to develop severe clinical signs with *M. haemofelis*.

Marked fluctuations in blood *M. haemofelis* copy numbers were seen in the current study at varying time points PI, but these were not as consistent or marked as the variation seen previously [7]. Long term cycling (up to around 50 DPI) of *M. haemofelis* copy number, characterized by regular increase and decrease in copy number over time, was previously documented in half of *M. haemofelis*-infected cats [7] whereas the current study failed to document similar cycling over this period in any of the nine *M. haemofelis*-infected cats monitored for this period. Convincing copy number cycling occurred in a few cats in the current study, but was documented later in the time course of infection. The reason for seeing less copy number cycling in the current study is not known. The same *M. haemofelis* isolate was used in both studies, although the current study cats tended to be younger. Doxycycline treatment was administered occasionally to some of the cats in the current study, which may be responsible for the large decreases in *M. haemofelis* copy numbers observed at those time points and/or resulted in a decreased tendency to cycle subsequent to treatment, but an absence of copy number cycling was also evident in most of the cats that did not receive doxycycline. Complex host–organism interactions are likely to be involved in copy number cycling and prediction of copy number cycling in an individual cat is probably not possible.

Several qPCR replicates were performed using the same DNA preparations extracted from cats in which the blood qPCR haemoplasma copy numbers were negative at the time that tissue samples were collected. This was done in order to maximize the accuracy of blood copy number determination in these cats as we did not want to assume that a single negative qPCR result reflected negative blood *M. haemofelis* status. Indeed we found that all of the cats initially negative on qPCR actually contained on average low *M. haemofelis* copy numbers in their blood, rather than being repeatedly negative (Table 2). Such a discrepancy could lead to an under-estimation of haemoplasma prevalence occurring in studies in which prevalence figures are based on single PCR results. It could be argued that replicates should also have been performed on DNA extracted from tissue samples yielding negative qPCR results. However our study was particularly interested in tissues containing higher *M. haemofelis* loads than blood levels, as these were putative tissue sites of *M. haemofelis* sequestration, so tissues yielding negative qPCR results were not further evaluated.

This study used a *M. haemofelis* qPCR assay duplexed with a 28S rDNA qPCR assay as an internal control. Demonstration of adequate 28S rDNA in samples confirmed the presence of amplifiable DNA and an absence of significant PCR inhibition in the PCR but results were not used to standardize haemoplasma copy numbers between cats or tissues. Standardization to an internal control which is associated with nucleated cells, such as 28S rDNA, would be very artificial, and lack of an appropriate control is a recognized problem for erythrocytic parasites such as haemoplasmas. We therefore reported haemoplasma copy numbers per ml of blood, or per weight of tissue, as has been done in previous studies [9,15,16].

Those cats which had blood and tissues collected at times of high and moderate *M. haemofelis* copy number tended to have fewer *M. haemofelis* copies in their tissues than was expected due to the tissue blood supply alone, with only the spleen and lung generally containing a greater number of *M. haemofelis* copies. It is surprising that most tissues contained fewer *M. haemofelis* copies than expected. This could arise due to compromise of the blood supply to those tissues at the time of tissue collection or reflect a problem with the calculation used to compare blood and tissue haemoplasma copy numbers. A proven method for comparing blood and tissue haemoplasma copy numbers has not been reported. Different amounts of starting material used for blood compared to tissues, and the fact that tissue samples unavoidably contain blood, complicate interpretation. We felt it very important to take the tissue's blood supply into account as we were particularly interested in determining whether haemoplasma organisms were concentrated within tissues rather than being present purely due to the tissue's blood supply. However the ratios reported in Table 2 must be regarded as estimates because, as mentioned previously, the blood supply of the different tissues is likely to vary which has not been taken into account due to lack of such data for feline organs. Additionally DNA extraction is unlikely to be equally efficient in all tissues, and the absence of an appropriate internal control housekeeping gene for erythrocytic organisms precludes standardization of results for the different tissues. Tissues were also collected immediately after barbiturate overdose which is likely to have affected blood volume distribution. An over- or under-estimation of blood supply to an individual organ tissue would lead to an abnormally low or high ratio being calculated for that tissue. It is possible that the high ratios calculated for the splenic samples in those cats with moderate or high blood *M. haemofelis* copy numbers arose partly due to an under-estimation of blood supply to splenic tissues since our calculations did not account for possible increased splenic blood volume as a result of barbiturate administration. Table 2 also shows the raw qPCR copy number obtained for each tissue so that these figures are also available for evaluation of tissue haemoplasma content.

Spleen and lung were most likely to contain a greater number of *M. haemofelis* copies compared with the tissue blood supply alone in cats with high or moderate numbers of blood *M. haemofelis* copies. Although this could arise due to calculation problems and an under-estimation of blood supply to these tissues, as discussed above in relation to the spleen, these tissues have been proposed as sites of haemoplasma sequestration [11]. Additionally electron microscopical studies [17] have shown phagocytosis of haemoplasma organisms by macrophages as well as pitting, in the spleen.

Collection of blood and tissues at times of putative copy number cycling allowed specific evaluation as to whether *M. haemofelis* sequestrates within tissues, since this and the subsequent release of organisms was proposed as a mechanism to mediate the large decreases and increases in blood copy numbers reported with *M. haemofelis* infection. Since the collection of tissues defined the end point of use of the cats in the current study due to euthanasia, it is impossible to confirm that tissues collected at times of low *M. haemofelis* copy number represented time points before which a large increase in *M. haemofelis* copy number was due to occur. Cats HF8 and HF6 both showed convincing regular increases and decreases in *M. haemofelis* copy number immediately before tissue collection, so it was felt these samples were those most likely to represent tissues collecting during a nadir of copy number cycling. Possible cycling was also reported in HF12. The tissues collected from these three cats often contained no detectable *M. haemofelis* copies with the few yielding positive qPCR results containing only low *M. haemofelis* copy numbers, albeit with often high ratios indicating that although low, these copy numbers represented greater numbers of *M. haemofelis* than were expected due to the tissue blood supply alone. Although such ratios could represent low level organism sequestration, it was anticipated that in order for sequestration to mediate an almost immediate 4–5 log increase in blood *M. haemofelis* copy number by tissue release of organisms, as has been proposed with copy number cycling figures, many more organisms would need to be present within sequestering organs than those found in the current study. The kinetics and marked increase in haemoplasma copy number seen in the blood during the immediate PI period confirms that rapid multiplication of organisms must be possible in infected cats. Therefore rapid multiplication may be responsible for the marked rapid increases in *M. haemofelis* copy number that can be seen in infected cats during cycling, rather than organism release from sequestration sites. Additionally we feel that the marked rapid decreases in *M. haemofelis* copy number recorded in some cats are likely mediated by rapid effective clearance of organisms from the blood and their subsequent destruction.

## Figures and Tables

**Table 1:** Summary of recipient cat characteristics, inoculations given and time of tissue sample collection.

Recipient Cat ID	Age	Sex	*M. haemofelis* copy number present in intravenous inoculum dose	Time post-infection of tissue collection (day)	Haemoplasma copy numbers in blood at time of tissue collection
HF2	7 months	MN	1.55 × 10^8^	149	Low
HF3	7 months	FE	1.55 × 10^8^	98	Moderate
HF4	7 months	MN	1.55 × 10^8^	177	Low
HF5	7 months	MN	1.55 × 10^8^	14	High
HF6	7 months	MN	1.55 × 10^8^	224	Low
HF7	7 months	FE	1.55 × 10^8^	98	Moderate
HF8	7 months	FE	1.55 × 10^8^	232	Low
HF9	7 months	FE	1.55 × 10^8^	104	Moderate
HF10	7 months	FE	1.55 × 10^8^	111	Moderate
HF11	7 months	MN	1.55 × 10^8^	18	High
HF12	7 months	MN	1.55 × 10^8^	167	Low
HF13	2 years	FE	3.87 × 10^7^	9	High
HF14	2 years	FE	7.21 × 10^7^	11	High

ID indicates identification; MN indicates male neutered, FE indicates female entire; Blood copy numbers were defined as being high if > 10^9^ copies/ml blood, moderate if between 10^7^ and 10^9^ copies/ml blood inclusive, and low if < 10^7^ copies/ml blood.

**Table 2:** Summary of qPCR copy number results for blood and tissue samples collected simultaneously from cats.

	Cat Identification							
	**HF5**	**HF11**	**HF13**	**HF14**	**HF3**	**HF7**	**HF9**	**HF10**
	qPCR copy number^a^ (ratio of tissue copy number to estimated copy number expected in tissue due to blood supply alone^b^)							
**In blood at time of tissue collection**	High: 2.64 × 10^9^	High: 1.30 × 10^10^	High: 1.63 × 10^9^	High: 4.01 × 10^9^	Moderate: 1.17 × 10^8^	Moderate: 7.2 × 10^7^	Moderate: 2.88 × 10^8^	Moderate: 2.51 × 10^8^
**Expected in tissue if a positive result was due to blood supply alone**^c^	43,631	264,233	26,866	66,115	1932	1190	4755	4139
**Tonsil**	9828 (0.23)	12,099 (0.05)	7,984 (0.3)	18,333 (0.28)	379 (0.2)	102 (0.09)	330 (0.07)	24 (0.01)
**Colon**	6052 (0.14)	63,790 (0.24)	2459 (0.09)	2295 (0.03)	51 (0.03)	117 (0.1)	177 (0.04)	180 (0.04)
**Submandibular salivary gland**	25,921 (0.59)	14,893 (0.06)	3726 (0.14)	27,781 (0.42)	500 (0.26)	330 (0.28)	1998 (0.42)	354 (0.09)
**Mesenteric lymph node**	3027 (0.07)	17,106 (0.06)	**146,475 (5.45)**	55,537 (0.84)	17 (0.01)	6 (0.01)	89 (0.02)	17 (0.004)
**Lung**	**313,827 (7.19)**	**2,507,367 (9.49)**	127,525 (4.75)	59,521 (0.9)	**10,533 (5.45)**	**17,106 (14.37)**	**24,187 (5.09)**	1148 (0.28)
**Colonic lymph node**	1071 (0.02)	18,333 (0.07)	9828 (0.37)	63,790 (0.96)	24 (0.01)	8 (0.01)	268 (0.06)	67 (0.02)
**Liver**	103,596 (2.37)	221,957 (0.84)	34,198 (1.27)	146,475 (2.22)	870 (0.45)	615 (0.52)	3727 (0.78)	870 (0.21)
**Bone marrow**	**273,226 (6.26)**	180,309 (0.68)	**168,241 (6.26)**	193,242 (2.92)	574 (0.3)	1071 (0.09)	2825 (0.59)	999 (0.24)
**Spleen**	**3,645,186 (83.55)**	**3,307,918 (12.52)**	**475,550 (17.7)**	**1,654,674 (25.03)**	**25,921 (13.42)**	1413 (1.19)	**45,116 (9.49)**	6051 (1.46)
**Kidney Cortex**	45,116 (1.03)	68,366 (0.26)	12,099 (0.45)	90,193 (1.36)	707 (0.37)	358 (0.3)	1071 (0.23)	287 (0.07)
**Kidney Medulla**	9170 (0.21)	73,269 (0.28)	8557 (0.32)	45,116 (0.68)	3027 (1.57)	2636 (2.22)	4916 (1.03)	154 (0.04)
**Mid-jejunum**	2459 (0.06)	10,533 (0.04)	4280 (0.16)	6052 (0.09)	31 (0.02)	36 (0.03)	190 (0.04)	125 (0.03)

	Cat Identification				
	**HF2**	**HF4**	**HF8**	**HF12**	**HF6**
	qPCR copy number^a^ (ratio of tissue copy number to estimated copy number expected in tissue due to blood supply alone^b^)				
**In blood at time of tissue collection**	Low: 1346^d^	Low: 54^d^	Low: 62^d^	Low: 152^d^	Low: 460
**Expected in tissue if a positive result was due to blood supply alone**^c^	2.2 × 10^−2^	8.9 × 10^−4^	1.02 × 10^−3^	2.5 × 10^−3^	7.9 × 10^−3^
**Tonsil**	Negative	**5.5 (6179.78)**	**2.41 (2362.75)**	Negative	Negative
**Colon**	**0.6 (27.27)**	Negative	Negative	Negative	Negative
**Submandibular salivary gland**	Negative	Negative	Negative	Negative	Negative
**Mesenteric lymph node**	Negative	Negative	Negative	Negative	Negative
**Lung**	Negative	**2.97 (3337.08)**	Negative	Negative	Negative
**Colonic lymph node**	**3.41 (155.00)**	Negative	Negative	Negative	Negative
**Liver**	Negative	Negative	Negative	Negative	**1.71 (216.46)**
**Bone marrow**	Negative	Negative	Negative	Negative	Negative
**Spleen**	**2.25 (102.27)**	Negative	Negative	**3.65 (1460.00)**	**1.21 (153.16)**
**Kidney Cortex**	Negative	Negative	Negative	Negative	Negative
**Kidney Medulla**	Negative	Negative	Negative	Negative	Negative
**Mid-jejunum**	Negative	Negative	Negative	Negative	Negative

a Figures indicate *M. haemofelis* copy number found on qPCR per ml of blood or per 0.5 mg of tissue.

b Tissues with ratios ≥ 5 are highlighted in bold.

c Calculated taking into account the amount of material used for the blood and tissue qPCR assays and the estimated blood volume of the tissues at time of the sampling.

d Average blood copy number was calculated by repeat qPCR haemoplasma analysis performed eight times using replicate aliquots of the same extracted DNA preparations to assess how repeatedly low or negative the copy numbers in these cats were to enable more informed interpretation of any low positive tissue qPCR results obtained in the same cats.
